# Complex Interventions Deserve Complex Evaluations: A Transdisciplinary Approach to Evaluation of a Preventive Personalized Medicine Intervention

**DOI:** 10.3389/fpubh.2022.793137

**Published:** 2022-02-04

**Authors:** Elise M. Garton, Serdar Savaş, Christopher Pell, Elena V. Syurina, Karien Stronks, Tomris Cesuroglu

**Affiliations:** ^1^Athena Institute, Faculty of Science, Vrije Universiteit Amsterdam, Amsterdam, Netherlands; ^2^Gentest Institute, Istanbul, Turkey; ^3^Amsterdam Institute for Global Health and Development, Amsterdam, Netherlands; ^4^Academic Medical Centre, University of Amsterdam, Amsterdam, Netherlands

**Keywords:** transdisciplinary research, realist evaluation, personalized healthcare, non-communicable diseases, preventive healthcare, program evaluation

## Abstract

Non-communicable diseases (NCDs) are the largest cause of disability and death globally. The human and financial costs of NCDs have raised questions of sustainability for many health systems. Personalized, preventive health interventions are an innovative way to address NCDs, but it is difficult to measure their effectiveness using standard evaluation methods. This article describes a novel approach to evaluation by coupling transdisciplinary methods with realist theory to design and pilot a health outcomes evaluation for a personalized medicine approach to NCD prevention in Istanbul, Turkey. Research and practice stakeholders contributed to study design, research questions, validation of results, and recommendations through interactive workshops, consistent dialogue, and reflection. They co-created a customized outcome measurement framework and recommendations that promote sustainability and continuous improvement of future evaluations. The participatory methods helped resolve the dichotomy between patient, practitioner, and researcher focus in the evaluation and improved stakeholders' data literacy. This research contributes to the body of evidence advocating for the use of non-standard methods such as transdisciplinary research to evaluate the effectiveness of complex interventions. The results of the pilot evaluation are also presented as a case study.

## Introduction

Non-communicable diseases (NCDs) account for 74% of deaths and 62% of disability-adjusted life years globally (1). Diseases including cancer, cardiovascular disease, diabetes, and chronic respiratory diseases, along with their risk factors such as an unhealthy diet, physical inactivity, and substance use, reduce people's life expectancy, quality of life, and economic productivity (2). In addition to the health impacts, the financial consequences of NCDs for individuals and their families—in terms of lost economic output and medical expenses—damage the local and national economies (3). These impacts are felt disproportionately by poor and vulnerable populations because they cannot recover easily from the physical and financial burdens of disease (4). When combined with an aging population and decreasing insurance premium revenues, the growing burden of disease due to NCDs is driving many health systems toward unsustainability (2, 3, 5).

Personalized medicine has been recognized as an approach to NCDs that contrasts with population-wide campaigns or individual treatment programs. Personalized medicine has many definitions; in this article it is used to describe a holistic approach to clinical practice using genomic, clinical, and lifestyle factors and involving all three dimensions of care to extend life and improve its quality (6–9). Preventive personalized medicine refers specifically to such an approach before the disease is diagnosed or even suspected. Preventive personalized medicine interventions increase motivation among patients and providers, provide a favorable benefit-risk ratio, and improve health outcomes (10). However, compared to population-level approaches, they have high initial costs and can be challenging to scale (10, 11).

More research is needed on the effectiveness of personalized medicine approaches that draw on genetic and lifestyle information. However, traditional forms of clinical research such as randomized control trials are not well-suited to such assessments. Although randomized control trials can prove a causal link between individual risk factors and the development of an NCD, they do not illuminate the mechanisms that account for such an effect. In addition, preventive interventions are generally more complex than single sets of exposures and outcomes. This necessitates the use of large, longitudinal cohort studies to evaluate personalized preventive interventions, but such studies face challenges related to cost, high variability between individuals, and the long follow-up time needed to determine and quantify health and disease outcomes (12, 13). These challenges are exacerbated when the personalized approach includes genomics, or the effects of individual genes on disease progression and prevalence, due to the rapid evolution of genomics research (12–14). This study challenges these traditional forms of assessing evidence using transdisciplinary research to design and pilot a measurement framework for a complex personalized health intervention.

Transdisciplinary research (TDR) uses participatory methods to co-create knowledge through reflection among all stakeholders, with the aim of going beyond scientific results to promote sustainable change (15). TDR is well-suited to assessing complex personalized health interventions for three main reasons. First, it creates space for the academic and experiential knowledge of researchers, practitioners, and consultees, all of whom provide valuable information and context for evaluation design. Second, it prioritizes sustainability and the real-life application of results to improve health. Finally, it emphasizes the importance of reflection and learning from the research process as well as the findings, which provides flexibility as the intervention and the assessment develop. These last two features of TDR also ensure that the project is beneficial to the researcher, the funder, and research participants.

Because of the integrative nature of TDR, a theoretical design of an outcome measurement framework is ineffective; it needs to be based on an existing case. As such, this research takes the form of a case study of a particular preventive personalized medicine intervention. The case study focuses on Gentest, the clinical practice arm of a program developed in 2005 at GENAR Institute for Public Health and Genomics Institute that aims to prevent common non-communicable diseases in a primary care setting (16). Gentest is based on the principles of 7K Medicine, named for seven words beginning with a “K” in Turkish: personalized, predictive, preventive, comprehensive, precise, evidence-based, and participatory (17). Gentest has been led since its inception by Dr. Serdar Savaş, a physician who previously worked for the Turkish Ministry of Health on national health reform and the World Health Organization Regional Office for Europe, and is currently implemented at Gentest Institute, a private clinic in Istanbul (16).

Gentest consultees—a term used rather than patients to emphasize the participatory nature of their involvement in their treatment—undergo an extensive lifestyle questionnaire, family and medical history assessments, genetic testing, microbiome analysis, body composition measurement, exercise and heart rate monitoring, and urine and blood tests. A team of physicians, dieticians, geneticists, and nurses use this information to create personalized goals, supplement and medication regimens, and detailed dietary and lifestyle plans based on a consultee's health priorities and his or her genetic and lifestyle-related risks of developing specific NCDs. The consultee's progress is monitored and updated through appointments with clinicians (17), repeated lab tests, and one-on-one support *via* WhatsApp from a dietician and physician. These practices aim to accomplish three goals: raise awareness of disease risk through information, change attitudes by creating a perception of individual vulnerability, and stimulate behavior change through follow-up programming and care (17).

Gentest is a private organization and all consultees pay for services out-of-pocket (17). Consultees are wealthy, middle-aged and older, and well-educated—they have a median age of 50, and 64% have a university degree as compared to 15% of the general Turkish population (18). In addition, Gentest occupies a unique space in the greater context of NCD prevalence and management in Turkey. There, NCDs account for 88% of deaths and 82% of disability-adjusted life years. Since 1990, those figures have risen by 33 and 54% respectively, indicating rapid growth of NCDs and inadequate prevention and treatment at the national level (1). Although the Turkish Ministry of Health has responded with population-level interventions, the health system's approach to NCDs remains reactive and rooted in secondary care treatment rather than prevention in primary care (2). Prevention of NCDs is considered outside the domain of primary care (19, 20).

Little is known about the impact of the 7K Medicine approach applied by Gentest on NCD prevention. Moreover, for this—and similar interventions—approaches to evaluation are inappropriate for practical and methodological reasons. Drawing on a TDR approach, this article describes the development of a measurement framework for the impact of 7K Medicine in health outcomes. Through this case study, the article explores the use of transdisciplinary methods for monitoring and evaluating complex interventions.

## Materials and Methods

### Theoretical Framework

**Figure 1:**
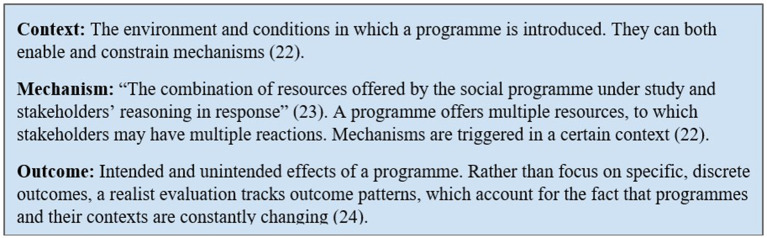
Key components of realist evaluation.

Because Gentest is an active clinical practice with a unique consultee population, this study leverages many elements of realist evaluation in its conceptual model and methods. Realist evaluation was developed by Tilley and Pawson in 1997 to assess real-life interventions differently from traditional scientific experiments. It is based on the assumptions that programs are embedded in social systems, require active engagement from stakeholders and participants, and exist in open systems that cannot be isolated or kept constant (21). Because of these circumstances, it is impossible to answer the question “does the program work?” in an evaluation, as observational evidence alone cannot establish causality (22). Instead, a realist evaluation asks, “what works for whom, under what circumstances, and how?” (23) and answers these questions through a process of “sense-making” between the context, mechanisms, and outcome patterns of a program (21). These three key components of a realist evaluation are defined in Figure 1.

**Figure 2:**
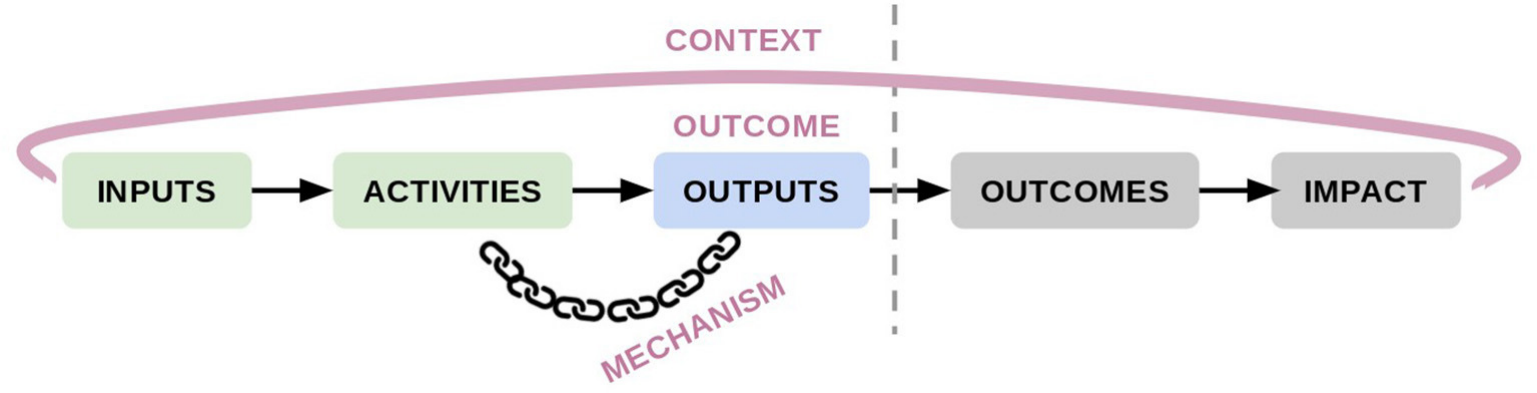
Conceptual framework for the study.

The context-mechanism-outcome framework of the realist evaluation was superimposed on the traditional logical framework (logframe) tool used for program evaluation to create the conceptual framework for this study, shown in Figure 2. The Supplementary Figure 4 and depicts operations and goals of Gentest mapped to the logframe.

Although the logframe's outcomes and impacts are included in the conceptual model, they are shown in gray because the time constraints of Gentest's implementation and this research do not allow for their evaluation. Thus, the outcomes of a realist evaluation are synonymous with the outputs of the logframe and are referred to hereafter as “outcomes.”

The use of realist evaluation helps account for strategic changes and fluidity over time in Gentest's implementation, lack of consistent and comprehensive data, and variability in intervention goals that would act as limitations in other, more linear evaluation frameworks. It also limits the urge to generalize the effectiveness of Gentest's intervention to other contexts and conditions (24, 25). The lack of generalizability does not invalidate this research, however, since the transdisciplinary design of an outcome measurement framework for these types of interventions offers transferable lessons.

### Transdisciplinary Design

The evaluation design and pilot used transdisciplinary methods with an emphasis on stakeholder involvement in every phase of the research. This was structured through four phases: research preparation, data collection, data analysis, and translation of results to practice. The key activity of each phase were interactive workshops in which stakeholders formally contributed to research design, validation, and recommendation-building with knowledge from their research and practice roles (Table 1). As the lead researcher worked full-time at Gentest Institute for the duration of the study, her daily interactions with and observations of Gentest staff, advisors, and consultees also served as scoping observations that guided data collection, analysis, and knowledge exchange.

**Table 1:** Study phases and key activities.

**Phase**	**Workshop**	**Date and location**	**Purpose**	**Methods**
Research preparation	Workshop 0: Indicator selection	Feb. 27, 2020; Istanbul	Identify potential outputs for pilot evaluation.	Document review, shared brainstorming
Data collection	Workshop 1: Research question formation	Apr. 30, 2020; Remote *via* Zoom	Determine research questions of the pilot based on available data.	Collaborative review of preliminary results
Data analysis	Workshop 2: Results validation	Jun. 2, 2020; Remote *via* Zoom	Review and lend context to pilot evaluation results.	Guessing game to challenge preconceptions, collaborative review of results
Results to practice	Workshop 3: Recommendation building	Jun. 11, 2020; Remote *via* Zoom	Based on pilot experience, recommend organizational and technical changes to improve future evaluations.	Shared brainstorming, issue mapping, recommendation mapping

Workshop attendees included Gentest's director and physician, Dr. Serdar Savaş, Gentest's research and development coordinator, a co-researcher developing a realist program theory of Gentest, and relevant Gentest staff including dieticians, geneticists, and a nurse. The first workshop took place at Gentest's office in Istanbul and remaining workshops were conducted remotely due to constraints related to COVID-19. Although comprehensive end-user participation is ideal in TDR research, consultees were not represented in the workshops. This decision is explained in more detail in section Limitations. While the primary goal of each workshop is described in Table 1, a secondary goal of all sessions was for stakeholders to share opinions and experiences with each other and the researcher to co-create knowledge about Gentest and its effectiveness. Workshops were analyzed through organized notetaking during and after the sessions, and workshops results were compared with relevant literature. All notes and workshop materials were shared with participants for member check.

### Mixed Methods Case Study Data Collection and Curation

The pilot of the outcome measurement framework was primarily quantitative and took the form of a longitudinal retrospective cohort study. Gentest has treated over 1200 consultees in the past decade, but electronic data collection formats were not standardized until mid-2017 and thus older longitudinal data could not be easily obtained from existing documents. To ensure accuracy, a sample of adult consultees who were actively followed up at the time of the research was selected by Gentest and are referred to in the study as “active consultees.” Active status was determined based on consultees' most recent appointment date and the presence of multiple Gentest encounters between September 2018 and June 2020. Seventy-five potential outcome variables including consultee demographics, body measurements, lab results, anthropometric measurements, medical history, and perceived improvement in 20 categories were identified in Workshop 0. Because it is impossible to measure outcomes of NCDs over a short time period, all variables were surrogate markers to indicate the progression of disease. Gentest employees reviewed multiple sources of patient documentation and manually entered the selected data into Microsoft Excel to create a study sample of 100 consultees with longitudinal data measured between 2009 and 2020, depending on when the consultee had his or her first Gentest encounter.

Quantitative data was pre-processed and analyzed using Python (primarily pandas, matplotlib, and statsmodels). “Time in days since first Gentest encounter” was chosen as the longitudinal axis rather than date to facilitate better comparison between consultees, and missing data was imputed under the assumption that trends in output variables were linear between imbalanced measurement dates. Outliers were identified using the Monte Carlo simulation method and reviewed with Gentest dieticians familiar with the consultees and with data collection workflows to determine if the outliers were potentially accurate, the result of manual data entry errors, or likely errors in the original documentation (26). In response, manual data entry errors were corrected and likely errors in original documentation were removed. Lastly, one consultee was excluded from the sample to limit the data range to 2014–2020, leaving a final sample size of 99 consultees, 15 static measurements per consultee, and 10353 total measurements unevenly distributed across 62 time-series repeated variables.

### Case Study Data Analysis

After pre-processing, stakeholders reviewed the consultee-specific trends of each variable in Workshop 1 and identified fourteen outcome variables for further analysis (Table 2). Stakeholders selected these outcome variables based on data availability, perceived data accuracy,^1^ perceived relevance to the consultee and to Gentest, and clarity of interventions used to influence the health outcome. Gentest attempts to improve consultees' magnesium, selenium, vitamin B12, and vitamin D primarily through nutrition advice and prescription of supplements, while they use a combination of lifestyle and dietary measures to improve other outcomes. Although there are other areas targeted for improvement, such as physical activity, sleep quality, and quality of sexual life, they are not monitored and recorded objectively and were excluded from the list of outcomes.

**Table 2:** Outcome and explanatory variables selected for analysis.

Lab results	HbA1c, High-sensitive CRP, Homocysteine, Magnesium, Selenium, Total: HDL cholesterol ratio, Triglyceride, Vitamin B12, Vitamin D
Body measurements	Body mass index (BMI), Body fat percentage, Waist: Height ratio
Anthropometrics	Systolic blood pressure, Diastolic blood pressure
Explanatory Variables	Education group, Sex

Education group and sex were chosen as explanatory variables to analyze alongside longitudinal trends, with higher education defined as a completion of a university degree or above. It is important to note that this threshold for higher and lower education is not representative of the general Turkish population as the Gentest consultees are relatively well-educated. A chi-square test for independence was conducted to ensure that education group and sex were not correlated among the sample population.

Gentest Institute team provided defined risk thresholds per variable (and in some case per sex and age group) to indicate whether consultees were considered clinically “at-risk” and therefore targeted for improvement. An analysis of these thresholds (Supplementary Material) suggests that they either align with or are stricter than common risk stratifications set by research and healthcare leaders such as the American Heart Association and Mayo Clinic. These thresholds were used as inclusion criteria for the consultee sample of each statistical model. Because of the personalized nature of the intervention, consultees considered at-risk or not at-risk for a particular health indicator did not necessarily receive the same treatment or lack of treatment. The risk stratification designates only if improvement of that indicator was a goal of the consultee's individualized treatment, however that treatment manifested.

Statistical analysis was conducted using linear mixed effects models with the consultee as a random effect and time (since first Gentest encounter), at-risk status, education group, and sex as fixed effects. This regression model was selected because of its compatibility with unbalanced, abnormally distributed data, its assessment of both slope and intercept differences between consultee groups, and because it will become increasingly accurate as Gentest collects more data for its existing and new consultees (27). However, it is important to note that statistical power was low due to the small study population; any test with a sample size under 50 was below the standard acceptance level of 0.8.

Eight configurations of the mixed linear effects model were run per output variable to answer five total research questions (Table 3).

**Table 3:** Co-designed research questions.

**#**	**Research question**	**Consultee sample**	**Fixed effects**
1	Do at-risk consultees experience changes in progression or outcomes of NCDs targeted by Gentest interventions?	At-risk consultees	Time
2	Is there a difference experienced by at-risk consultees in progression or outcomes of NCDS targeted by Gentest interventions as compared to not at-risk consultees?	All consultees	Risk status, Time
3	Is there a difference in changes experienced by high and lower-educated consultees in progression or outcomes of NCDs targeted by Gentest interventions?	At-risk consultees	Time, Education group
4	Is there a difference in change experienced by male and female consultees in progression or outcomes of NCDs targeted by Gentest interventions?	At-risk consultees	Time, Sex
5	Do men, women, lower educated, or higher educated Gentest consultees experience changes in progression or outcomes of NCDs targeted by Gentest interventions?	At-risk lower educated consultees	Time
		At-risk higher educated consultees	Time
		At-risk male consultees	Time
		At-risk female consultees	Time

The different model configurations were conducted to provide Gentest with as much information as possible about the health progression and outcomes of its consultees, especially those considered at-risk for a certain disease and therefore targeted by personalized interventions. Although education group and sex were not correlated based on results of a chi-squared test for independence, they could not be analyzed together as sample sizes were too small. Results were assessed for both progression and baseline measurement of each variable since both are clinically relevant.

The quantitative analysis was supplemented by a brief qualitative assessment of answers provided by a separate sample of 156 consultees (with considerable overlap with the primary consultee sample) to the question “Why did you come to Gentest?” asked at their first Gentest encounter. This data was obtained by loading historical, non-longitudinal data for 176 consultees. This additional analysis aimed to identify congruence between consultee goals and the goals of Gentest practitioners as indicated by their variable selection. Responses were translated from Turkish to English using Python's Google Translate API and unclear translations were validated with a native Turkish speaker. Translated responses were categorized in two rounds using 14 code categories. Following the coding, the prevalence of code categories was compared to better understand Gentest consultees' motivation.

## Results

This section presents the detailed results of six of the eight linear mixed effects models used in the case study, explains the findings of all eight models, and introduces the qualitative results before presenting the joint interpretation of these results formed in Workshop 2. Synthesizes results of the workshop are available in Supplementary Figure 6.

### Case Study Results

The study population of 99 consultees included 56 men and 43 women. 62 consultees were categorized as higher educated (38 bachelor's degrees, 22 master's degrees, and 2 doctoral degrees), while 32 were categorized as lower educated (20 with a high school education and 11 with below a high school education). Five consultees were not included in education-stratified analysis as their education level was unknown. The majority (69) of consultees lived in Istanbul, with Bebek as the most common municipality (*n* = 11). All remaining consultees lived in other cities in Turkey at their time of enrollment at Gentest. Consultees ranged in age from 29 to 93, with a median age of 50. Documented existing chronic diseases or behaviors of consultees are shown in Table 4.

**Table 4:** Comorbidities of study population.

Anxiety	14	82	3
Cancer	5	90	4
Depression	16	80	3
Diabetes	23	74	3
High cholesterol	32	65	2
Hypertension	22	74	3
Hypertriglyceridemia	14	83	2
Smoking	30	67	2

Table 5 contains the results of research questions 1, 2, and 5 as listed in Table 3. The results of questions 3 and 4 are included in Supplementary Table 8. Each outcome and fixed-effect combination reports the sample size (*n* and %), slope of the mean trajectory (β), 95% confidence interval, and significance (p) at α = 0,05 of the difference in trajectory as compared to 0 or no change for each population group. *P*-values with asterisks indicate statistical significance, and “NA” indicates that the model could not converge and reliable results were not possible. β values are low across all models because the longitudinal axis is measured in days. However, even in cases with statistically significant improvement, the rate of change of improvement is still small for nearly all outcomes, as expected for surrogate markers of NCDs and long-term health. Although there were 99 consultees included in the total sample, consultees were only included in the statistical analysis of each outcome if they had two or more measurements for that outcome. This explains the variation in sample sizes.

**Table 5:** Selected results of quantitative analysis.

**Risk group**	***n***	**%**	**β** **Rate of change**		**95%CI**	***p***
HbA1c						
Difference between at-risk and not at-risk			88	100%	–	–	0.000*
At-risk sample	All at-risk	13	100%	−0.001	−0.001 − −0.000	0.011*
	Lower educated	5	38%	NA	NA	NA
	Higher educated	8	62%	< -0.001	−0.001 − −0.001	0.524
	Male	8	62%	−0.001	−0.001 – < −0.001	0.003*
	Female	5	38%	−0.001	−0.002 − −0.001	0.000*
Triglyceride						
Difference between at-risk and not at-risk			89	100%	–	–	0.003*
At-risk sample	All at-risk	25	100%	−0.053	−0.095 − −0.010	0.016*
	Lower educated	8	32%	−0.052	−0.100 − −0.004	0.034*
	Higher educated	17	68%	−0.057	−0.013 – 0.015	0.119
	Male	20	80%	−0.052	−0.100 − −0.005	0.031*
	Female	5	20%	−0.065	−0.016 – 0.003	0.179
Homocysteine						
Difference between at-risk and not at-risk			88	100%	–	–	0.426
At-risk sample	All at-risk	80	100%	−0.002	−0.003 – 0.000	0.013*
	Lower educated	30	38%	−0.002	−0.003 − −0.000	0.011*
	Higher educated	50	63%	−0.001	−0.003 − −0.000	0.127
	Male	52	65%	−0.002	−0.003 − −0.000	0.009*
	Female	28	35%	−0.001	−0.003 – 0.001	0.259
Magnesium						
Difference between at-risk and not at-risk			86	100%	–	–	0.397
At-risk sample	All at-risk	65	100%	0.000	−0.000–0.000	0.303
	Lower educated	24	37%	< -0,001	−0.000 – 0.000	0.522
	Higher educated	41	63%	< -0,001	−0.000–0.000	0.501
	Male	45	69%	< -0,001	−0.000 – 0.000	0.430
	Female	20	31%	< -0,001	−0.000 – 0.000	0.766
Selenium						
Difference between at-risk and not at-risk			78	100%	–	–	0.005*
At-risk sample	All at-risk	38	100%	0.039	0.018 – 0.061	0.000*
	Lower educated	13	34%	NA	NA	NA
	Higher educated	25	66%	0.063	0.036 – 0.090	<0.001*
	Male	24	63%	0.053	0.028 – 0.078	<0.001*
	Female	14	37%	0.023	−0.016 – 0.072	0.254
Vitamin B12						
Difference between at-risk and not at-risk			88	100%	–	–	0.782
At-risk sample	All at-risk	28	100%	0.079	−0.008 – 0.236	0.322
	Lower educated	7	25%	0.022	−0.121 – 0.078	0.671
	Higher educated	21	75%	0.358	0.097 – 0.619	0.007*
	Male	17	61%	0.417	0.112 – 0.723	0.007*
	Female	11	39%	−0.035	−0.217 – 0.146	0.702
Vitamin D						
Difference between at-risk and not at-risk			88	100%	–	–	0.731
At-risk sample	All at-risk	61	100%	0.012	0.005 – 0.018	<0.001**
	Lower educated	21	34%	−0.002	−0.010 – 0.014	0.730
	Higher educated	40	66%	0.015	0.008 – 0.022	<0.001
	Male	39	64%	0.016	0.008 – 0.024	<0.001*
	Female	22	36%	0.002	−0.006 – 0.011	0.613
High-sensitive CRP						
Difference between at-risk and not at-risk			83	100%	–	–	0.002*
At-risk sample	All at-risk	28	100%	−0.001	−0.002 – 0.001	0.406
	Lower educated	12	43%	−0.003	−0.006 – 0.001	0.168
	Higher educated	16	57%	0.000	−0.002 – 0.002	0.938
	Male	19	68%	−0.001	−0.002 – 0.001	0.354
	Female	9	32%	−0.001	−0.005 – 0.003	0.644
Total:HDL cholesterol ratio						
Difference between at-risk and not at-risk			88	100%	–	–	0.018*
At-risk sample	All at-risk	57	100%	<0.001	−0.001 −0.000	0.032*
	Lower educated	18	32%	< -0.001	−0.001 – 0.000	0.007*
	Higher educated	39	68%	< -0.001	−0.001 – 0.000	0.402
	Male	40	70%	<0.001	−0.001 − −0.000	0.032*
	Female	17	30%	< -0.001	−0.001 – 0.001	0.806
BMI						
Difference between at-risk and not at-risk			88	100%	–	–	0.050
At-risk sample	All at-risk	43	100%	−0.001	−0.002 − −0.000	0.008*
	Lower educated	20	47%	−0.002	−0.003 − 0.001	0.003*
	Higher educated	23	53%	−0.001	−0.003 – 0.000	0.165
	Male	36	84%	−0.001	−0.002 − −0.000	0.009*
	Female	7	16%	−0.007	−0.014 – 0.000	0.064
Body fat %						
Difference between at-risk and not at-risk			88	100%	–	–	0.492
At-risk sample	All at-risk	76	100%	−0.001	−0.001 – 0.000	0.182
	Lower educated	31	41%	<0.001	−0.001 – 0.001	0.959
	Higher educated	45	59%	−0.001	−0.002 – 0.000	0.118
	Male	49	64%	< -0.001	−0.001 – 0.000	0.361
	Female	27	36%	−0.001	−0.003 – 0.001	0.273
Waist:height ratio						
Difference between at-risk and not at-risk			85	100%	–		0.271
At-risk sample	All at-risk	76	100%	<0.001		0.128
	Lower educated	31	41%	< -0.001	−0.000 − −0.000	0.013*
	Higher educated	45	59%	< -0.001	−0.000 – 0.000	0.778
	Male	49	64%	< -0.001	−0.000 – 0.000	0.137
	Female	27	36%	< -0.001	−0.000 – 0.000	0.518
Systolic blood pressure						
Difference between at-risk and not at-risk			88	100%	–	–	0.056
At-risk sample	All at-risk	39	100%	−0.001	−0.008 – 0.009	0.734
	Lower educated	17	44%	0.001	−0.008 – 0.009	0.855
	Higher educated	22	56%	−0.004	−0.015 – 0.006	0.421
	Male	26	67%	−0.002	−0.011 – 0.006	0.565
	Female	13	33%	NA	NA	NA
Diastolic blood pressure						
Difference between at-risk and not at-risk			87	100%	–	–	0.006*
At-risk sample	All at-risk	21	100%	−0.009	−0.0014 – 0.003	0.003*
	Lower educated	9	43%	−0.009	−0.010 − −0.003	<0.001*
	Higher educated	12	57%	−0.010	−0.017 − −0.003	0.005*
	Male	16	76%	−0.001	−0.016 − 0.003	0.006*
	Female	5	24%	−0.009	−0.024 – 0.005	0.197

The overall results for the at-risk population, answering research question 1, show significant improvement in eight of fourteen outcomes: HbA1c, triglyceride, homocysteine, selenium, vitamin D, BMI, total-to-HDL cholesterol ratio, and diastolic blood pressure. When the at-risk population was compared with the not at-risk population to answer research question 2, there were seven outcomes with significant differences in trends. In all cases, the at-risk population improved more than the not at-risk population. All other outcomes either stayed stable or improved among the not at-risk population except for HS-CRP, which worsened over time.

Education group was added as a fixed effect to answer research question 5. This analysis showed that lower educated consultees improved in six outcomes: triglyceride, homocysteine, BMI, waist-to-height ratio, total-to-HDL cholesterol ratio, and diastolic blood pressure. Higher educated consultees improved in five outcomes: triglyceride, selenium, vitamin B12, vitamin D, and diastolic blood pressure. When education groups were compared directly to answer research question 3, there were three outcomes with significant differences. Lower educated consultees had higher baseline waist-to-height ratios, indicating greater risk. Lower educated consultees showed comparatively more improvement in BMI than their higher educated counterparts, but they showed significantly less improvement in vitamin B12 levels.

Similarly, sex was added as a fixed effect to continue answering research question 5. This analysis demonstrated that men improved in nine outcomes: HbA1c, triglyceride, homocysteine, selenium, vitamin B12, vitamin D, BMI, total-to-HDL cholesterol ratio, and diastolic blood pressure. Women, however, improved in only one outcome, HbA1c. When men and women were compared directly to answer research question 4, there were three differences in baseline risk and two differences in improvement trends. Men had higher-risk baseline homocysteine levels and lower-risk baseline BMI and waist-to-height ratios, although both body measurement differences were expected because risk thresholds vary by sex for these outcomes. Men also showed comparatively more improvement than women in selenium levels. Women showed comparatively more improvement than men in BMI, but it is important to note that their non-comparative rate of change for BMI was insignificant, making this finding less meaningful. The comparative results to answer research questions 3 and 4 are presented in detail in the Supplementary Material.

**Figure 3:**
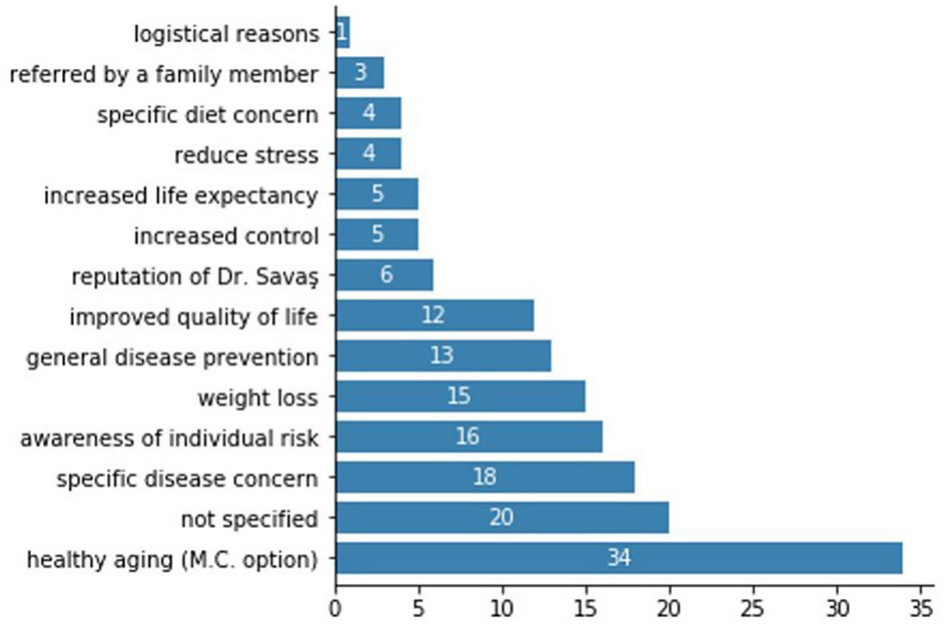
Categorized responses of new consultees when asked “Why did you come to Gentest?.”

Finally, the qualitative analysis of new consultee responses to the question “Why did you come to Gentest?” yielded the categorizations presented in Figure 3. The two most common categories, “healthy aging” and “not specified” were presented as multiple-choice options to some but not all consultees, so their high prevalence cannot be directly compared with other answers, which were documented as free response. Otherwise, the most common responses were concern about a specific disease (often an existing diagnosis or a disease prevalent in the consultee's family), awareness of individual risk, and weight loss.

### Integrated Review of Results

Gentest stakeholders reviewed the graphs of quantitative results in Workshop 2 and discussed each outcome trend in detail. Because a total of 112 outcome and fixed effect combinations were tested, this section outlines the notable patterns identified in the workshop rather than analyze the result of each model.

First, there was no clear pattern in differences in improvement between supplement-based outcomes (magnesium, selenium, vitamin B12, and vitamin D) and other outcomes that required a combination of dietary and lifestyle changes. Although Gentest stakeholders expected some improvement among all outcome variables, they expected more improvement among the supplement-based outcomes because they seem more straightforward to control and require a less-personalized regimen. Stakeholders reflected that the lack of improvement in magnesium and vitamin B12 levels was likely due to poor supplement compliance and the need for control of additional factors alongside supplements to improve these results.

Secondly, Gentest stakeholders discussed the mixed findings relating to education level and health improvement, particularly the fact that many lower education consultees saw greater improvement than higher educated consultees. Stakeholders suggested that the unique Gentest population and context accounted for these patterns. All consultees are affluent, and their high socio-economic status may negate the impact of their comparatively low education level. The intense personal support that Gentest provides may also reduce the role that education plays in a consultee's health improvement. In addition, many lower educated consultees share a household with higher educated consultees, and these dynamics may affect their behavior and compliance with Gentest's recommendations. Several Gentest employees also posited that higher educated consultees may have poorer compliance with dietary and lifestyle recommendations because they seem overconfident in their own judgment and less willing to listen to others' advice.

Thirdly, Gentest stakeholders reflected on the lack of statistically significant improvement among women. Although the small sample size may partially explain these findings (56 men and 43 women, with fewer at-risk women than men for nearly every outcome tested), Gentest stakeholders were initially surprised by these results. However, they quickly attributed the lack of improvement to women's unilateral focus on weight loss. They suggested this focus makes women less motivated to improve invisible indicators of health, such as lab results. Based on observations and discussions with Gentest employees, the lack of improvement among women may also be related to the unique gender dynamics at Gentest and the impact they have on consultees' behavioral decisions. Although Dr. Savaş is male, all other consultee-facing staff are women, and as one dietician mused, “woman to woman communication can be difficult.”

Finally, a closer analysis of consultees' responses to the question, “Why did you come to Gentest?” does not support the hypothesis that women are focused only on weight loss. Only 15 of 156 consultees cited weight loss as their primary reason for seeking Gentest treatment, and of those 15, just 6 (40%) were women. Women's responses more often fell into the categories of “specific disease concern” (*n* = 11), “general disease prevention” (*n* = 9), “improved quality of life” (*n* = 8), “awareness of individual risk” (*n* = 8), and the multiple-choice category of “healthy aging” (*n* = 19). This is certainly not conclusive evidence as weight loss is likely a component of those other responses for many women, but it supports the need for more research and involvement of female consultees to understand their motivations and the gender dynamics at Gentest.

### Outcome Measurement Framework

Based on a combination of the statistical results, the discussions in all four workshops, observations of Gentest's practice that indicated their capacity for changes to data collection and curation, and the analysis of consultee motivations, an outcome measurement framework was developed for Gentest (Table 6). These suggested outcome and explanatory variables are accompanied by 56 detailed organizational and technical recommendations that will help Gentest improve future evaluations. Indicators that were not included in the pilot but added to the framework are italicized, and their justification briefly explained below.

**Table 6:** Outcome and explanatory variables selected for measurement framework.

Lab results	HbA1c, Homocysteine, Magnesium, Selenium, *Testosterone*, Total: HDL cholesterol ratio, Triglyceride, Vitamin B12, Vitamin D
Body measurements	BMI, Body fat percentage, *Muscle mass percentage*, Waist: Height ratio
Anthropometrics	Diastolic blood pressure
NCDs	***Individual disease risk scores calculated by Gentest, Smoking status***
Perception	***Lifestyle progress ratings, Dietary progress ratings (average), Quality of life, Sleep quality, Sexual life quality, Supplement compliance***
Explanatory variables	***Appointment attendance***, Education, ***Enrolment duration, Genetic risk scores, Pre-existing conditions***, Sex, ***Shared household, Smoking status, Supplement compliance***

First, testosterone was added based on feedback from Gentest practitioners, who stated that aging male consultees compose the majority of Gentest's business and are often concerned about sexual performance as well as overall performance. Second, muscle mass percentage was added to reconcile inconsistent findings in risk assessment and improvement in BMI and body fat percentage. The additional indicator should help Gentest refine its risk stratification. Third, Gentest currently calculates individual disease risk scores using an algorithm based on the consultee's genetic, lifestyle, body measurements, and clinical data; this should be included first to assess its accuracy and later as a dependent variable. Finally, all newly added explanatory variables were mentioned by Gentest practitioners during the workshops as hypotheses to explain differences in improvement of outcomes between consultees.

All other newly added indicators were identified in Workshop 0 as important variables and are currently monitored by Gentest, but not in a standardized way that facilitates statistical analysis. Implementation of technical recommendations will allow these to be measured in the future. For example, consultees are currently asked about their sleep quality at each encounter, but going forward they will be asked to fill out a standardized short form to compare responses over time.

While the framework and the recommendations are Gentest-specific as expected in a realist evaluation, the discussion and lessons learned from the process of refining this framework are more generalizable and are explored in Section Discussion.

## Discussion

The discussion first reflects on the use of transdisciplinary methods and realist evaluation for the creation of an intervention evaluation, first at Gentest specifically and then more broadly. It then presents limitations of the study and ends with a conclusion to this research.

### Process Evaluation and Reflection—Gentest

Throughout the development and pilot of the outcome measurement framework, TDR and realist theory improved collaboration between researcher and participants, lent valuable contextual explanations to the pilot results, and facilitated an ongoing learning process between the researcher and stakeholders. There are two main areas in which these methods proved especially valuable and interesting compared to a more traditional evaluation approach.

First, the consistent sharing of knowledge and discussions about the goals of Gentest's practitioners and consultees led to a change in the definition of improvement of health outcomes. The pilot evaluation used Gentest-defined thresholds to identify and track the progress of “at-risk” consultees for each outcome indicator. However, upon reflection, stakeholders recognized that such a population-level approach—standard in most traditional evaluations—does not make sense for Gentest's highly personalized practice. For example, a consultee may prioritize weight loss, but clinical evidence suggests the consultee is at risk for diabetes, so the practitioner chooses to focus on reduction of HbA1c along with weight loss. However, the evaluator assesses the consultee's progress in weight loss and HbA1c, but also in several other outcomes that did not match the consultee or practitioner's priorities and were not the aim of the intervention.

This incongruence between consultee demand, medical need as identified by the practitioner, and researcher focus is common in many healthcare settings. After discussions and reflections enabled by TDR methods, Gentest stakeholders decided to use the pre-defined risk thresholds only as guidelines and instead assess risk on an individual, per-outcome basis. Evaluations of population-wide improvement will be driven by individual consultees' at-risk statuses so that Gentest's effectiveness can still be objectively measured while enabling a personalized and participatory approach toward risk assessment.

Secondly, the consistent involvement of Gentest stakeholders throughout the study increased the sustainability of the research and Gentest's ability to implement the findings. Prior to the study, Gentest practitioners had little awareness of what data they produced or how it could be used to make their work easier or help their consultees. As a result, many documentation practices were not data-friendly, such as heavy use of Microsoft Word, exclusive use of consultee-friendly images and icons in place of numerical data, duplication of information across platforms and sources, and lack of a consistent consultee identifier. Through their participation in this study, stakeholders better understand the impacts of these practices beyond their immediate job role and increased their accountability.

This shift in data literacy culminated in Workshop 3, when Gentest stakeholders created recommendations to improve indicator selection, data collection, data curation, and strategy based on their experiences and challenges during the study. Because these recommendations were informed by operational needs, Gentest can feasibly implement them while maintaining their commitment to consultee care. This co-creation of ideas and knowledge from the researcher and practitioners is a primary goal of TDR and what makes a TDR-designed evaluation more sustainable than a traditional alternative.

### Process Evaluation and Reflection—General

As a realist evaluation and pilot, many of the lessons learned and recommendations developed in this study are specific to Gentest Institute and its implementation of 7K Medicine. However, there are some considerations that can be applied to evaluation design or intervention for other complex health interventions.

First, this study points to the importance but also the challenge of knowledge integration between patients, practitioners, and researchers in evaluations of health interventions. The knowledge of all three groups is pivotal to bridge the gaps between patient demand, practitioner assessment of need, and researcher focus as explained in section Process Evaluation and Reflection—Gentest. However, knowledge integration is accompanied by inherent conflicts over who defines the goals and priorities of the intervention and the evaluation (28). This cannot and should not be avoided in TDR because these conflicts help to facilitate understanding (15). Instead, researchers and stakeholders must maintain awareness of this challenge, accept it as a meaningful addition to the research process, and reflect on it together in all phases of the evaluation.

Secondly, this study demonstrates that complex interventions deserve complex evaluations. The variability and imbalance of data, importance of context, and development of the program over time require explanations and validation from a variety of stakeholders, and the personalized and participatory principles of Gentest and 7K Medicine require a recognition of individual participants in both qualitative data collection and quantitative analysis. This is necessary to identify health progression and outcomes, but also to identify a causal connection between the activities and mechanisms of the intervention and its outcomes. The combination of TDR and realist evaluation were effective in this case study because they helped recognize and incorporate the unique characteristics of Gentest into the outcome measurement framework and pilot evaluation.

Finally, complex interventions are constantly evolving based on evidence and need, and the methods used to evaluate them should do the same. Sustainability of a complex evaluation does not mean ensuring that it can be repeated exactly the same way in the future, but rather that the right stakeholders have the tools and knowledge to improve future evaluations as they improve their interventions (29).

### Limitations

Because Gentest is an active clinical practice that has developed over the past decade, there were challenges in availability and quality of consultee data. First, the small study population (*n* = 99) limits the statistical power of the study. Second, missing data, inaccurately entered or stored data, and incomparable data between time periods were common. Third, although Gentest stakeholders provided clinical and contextual justification for their selection of outcome variables and thresholds, there was potential for bias in their selection as they naturally hoped their choices would demonstrate Gentest's effectiveness in improving health outcomes. Lastly, due to the convenience sampling of active consultees to accommodate comparable data collection, the findings serve as an evaluation only of Gentest's impact on the health of this group, not on former or lapsed consultees. This sampling method introduces bias as active consultees are likely more compliant and therefore likely to see greater improvement than lapsed consultees as they chose to exist the intervention

These limitations are by-products of the methodology. First, a realist evaluation expects and accepts the use of real-life, “messy” data, and the statistical methods chosen were also appropriate for this type of data. Second, the inclusion of Gentest staff as key stakeholders and creators of the research questions purposely introduced their bias into the variable selection. Although this seems to contradict traditional evaluation best practices, it would also be unfair to evaluate Gentest on outcomes that it does not aim to improve. Both limitations can be mitigated in future evaluations through the implementation of recommendations that aim to improve data quality and quantity and therefore provide more potential outcome variables. In addition, the outcome measurement framework resulting from the research is specific to Gentest, as is the aim of a realist evaluation, but the methods used to develop that framework are generalizable to other contexts.

The lack of consultee participation is another limitation regarding the TDR methods and participatory aims of the study. Although consultee appointments were observed and the responses to the question “Why did you come to Gentest” were analyzed to better understand consultees' motives, consultees were not included directly due to logistical constraints related to the COVID-19 pandemic. This question is addressed by research undertaken separately with Gentest consultees.

## Conclusion

An outcome measurement framework for an innovative personalized medicine model (Gentest Institute) was developed and piloted. The use of a transdisciplinary approach with realist evaluation ensures that this Gentest-specific framework is feasible to implement and sustainable. As a realist evaluation, this study cannot prove the general effectiveness of TDR, but it illustrates how non-standard research methods, such as TDR, can be applied to evaluate the effectiveness of complex interventions. More insight into the utility of these interventions will inspire adoption of such interventions, which will help achieve the ultimate goal of reducing the prevalence and impact of non-communicable diseases.

## Data Availability Statement

The datasets presented in this article are not readily available because Ethics Approval and Gentest Institute Policy state no individual participant results will be published or accessed by anyone other than the research team. Requests to access the datasets should be directed to Elise Garton, egarton@alumni.nd.edu.

## Ethics Statement

The studies involving human participants were reviewed and approved by Uskudar University Ethical Review Board, Uskudar University, Istanbul, Turkey. The patients/participants provided their written informed consent to participate in this study (30).

## Author Contributions

TC served as the main supervisor and EG oversaw execution. EG and SS had full access to all the data in the study and took responsibility for the integrity of the data. EG had a primary role in data collection and analysis, conceptualization, and writing of this manuscript. TC had a primary role in editing of this manuscript and a secondary role in data collection and conceptualization. SS had a secondary role in data collection and provided technical and organizational support. CP, ES, and KS advised on theoretical and analysis methods and had a role in conceptualization and editing of this manuscript. All authors have read and approved the final manuscript.

## Funding

This study is a part of the Developing a research agenda on primary health care with the 7K Medicine approach project, which received funding from the Amsterdam Public Health's Global Health Program—Promotion of Collaboration 2019. It is a collaboration between Athena Institute (VU Amsterdam); department of Public Health at Amsterdam UMC, location AMC; Amsterdam Institute for Global Health and Development (AIGHD); and Gentest Institute. Gentest Institute provided in-kind support to the study in various forms. For EG, this work fulfils the second-year thesis requirements of the Global Health Research Master program at Vrije Universiteit Amsterdam.

## Conflict of Interest

SS and TC are affiliated with the Gentest Institute, where the research took place. At the time of the study, EG was an intern at Gentest Institute as part of her training in Research Master in Global Health, Vrije Universiteit Amsterdam.

The remaining authors declare that the research was conducted in the absence of any commercial or financial relationships that could be construed as a potential conflict of interest.

## Publisher's Note

All claims expressed in this article are solely those of the authors and do not necessarily represent those of their affiliated organizations, or those of the publisher, the editors and the reviewers. Any product that may be evaluated in this article, or claim that may be made by its manufacturer, is not guaranteed or endorsed by the publisher.

## Footnotes

^1^“Perceived data accuracy” refers to the Gentest employees' assessment of the accuracy of the patient data based on their experiences collecting that data. For example, standard lab tests resulted from the same laboratory over the study time period were considered accurate, while body measurements conducted on multiple devices that were not comparable were considered not accurate for the purposes of longitudinal research and were not selected as outcome variables.
