# Supplementary material for: Complex Interventions Deserve Complex Evaluations: A Transdisciplinary Approach to Evaluation of a Preventive Personalized Medicine Intervention
Source: Front Public Health. 2022 Feb 4;10:793137. doi: 10.3389/fpubh.2022.793137 (PMC8854757; doi:10.3389/fpubh.2022.793137)
Supplement: Supplementary file 1 [file Data_Sheet_1.pdf]

DATA SHEET S1. Synthesized results of qualitative workshops

The 56 recommendations listed below are the synthesized results of Workshops 1-3, conducted in spring 2020. Challenges and recommendations were identified either by myself, the Gentest team in workshops #2 and #3 (June 2020), the research team, and current and former Gentest staff in informal discussions. In most cases, recommendations were the product of both Gentest experience and research findings.

Many of these recommendations may have already been implemented by Gentest, but not before the data used for the research was obtained. Others might not be officially implemented at Gentest but are often already used in practice, and just need to be acknowledged and standardized.

The **Programme Evaluation Map – Full** contains all recommendations, organized and analyzed. Along with the map, a detailed **Recommendations Table** was provided to Gentest staff that included more details about the challenge that inspired the recommendation, the recommendation in full, the suggested prioritization, and the explanation and/or notes for that recommendation. In some cases, this column also contained specific technical details for implementing the recommendation.

PROGRAMME EVALUATION

INDICATOR SELECTION

OUTCOME SELECTION

1. Re-calculate the risk of diseases relevant to the patient at regular intervals
2. Measure other indicators of specific diseases when possible
3. Add quality of life, quality of sleep, and quality of sexual life as outcomes

OUTPUT SELECTION

4. Risk should be determined at an individual level
5. Identify and share “guideline” thresholds per indicator
6. Ensure that Radar is explained and interpreted as the patient’s personalised progress
7. Reconsider the list of categories included in Radar
8. Identify and increase tracking of exercise-related metrics
9. Exclude HS-CRP

DATA COLLECTION

INTERNALLY-PRODUCED: ORGANISATIONAL

10. Assess outcomes mentioned in #3 every 2-3 months using online short forms
11. Define standards for Radar scoring
12. Document justification for Radar scores assigned
13. Assess Radar for patients every 2-3 months
14. Collect information about only current, diagnosed health conditions
15. Identify and document relevant work/home environmental factors
16. Document whether a patient lives in a household with another Gentest patient
17. Collect the reason for coming to Gentest as a free-text response

INTERNALLY-PRODUCED: TECHNICAL

18. Use MS excel and multiple-choice answers or cell validation
19. Document Gentest ID consistently
20. Document the date that all data or information was retrieved
21. Conduct an audit of InfusionSoft fields and define which fields are used for what purpose
22. Add a data field to indicate whether or not an output is “targeted” per patient
23. Choose a single method for body measurements
24. Document body measurements in a single Excel sheet
25. Standardise the format and content of dieticians’ interview notes
26. Document supplements prescribed regimens and compliance
27. Document and date medication use, dosage, and compliance
28. Radar results should be tracked numerically in a single excel file
29. Track encounter dates and types
30. Do not double-document recommendations or regimens
31. Do not leave fields blank in the VTF
32. Document a patient’s preferred language
33. Review the list of fields that could not be loaded into the historical database and update the Bilgi Formu template accordingly

EXTERNALLY-PRODUCED

34. Request results from external companies in data-friendly formats
35. Consider repeating the BEBIS analysis
36. Track exercise indicators selected in #8 using a shareable mobile application or device

DATA CURATION

ORGANISATIONAL

37. Document lack of monitoring during spring 2020 due to COVID-19
38. Determine strategy for dealing with data from unique external labs
39. Review the genetic risk score algorithms for accuracy

TECHNICAL

40. Ensure all patient information is uploaded into InfusionSoft
41. Conduct regular InfusionSoft audits
42. Outline a process for efficient document transfer to InfusionSoft
43. Define a process in which all proposed documentation changes are reviewed from a technical perspective
44. Define patient document naming conventions
45. Make algorithms and formulas for things like risk score calculations transparent
46. Use scripts created by Elise
47. If Gentest changes its primary lab partner from Ozel Ahenk, review the lab-related scripts
48. Document patient "drop-outs"

DATA ANALYSIS

METHODOLOGY + STRATEGY

49. Select a significance level for statistical results
50. Apply explanations for inaccurate data consistently
51. Apply statistical methods consistently
52. Assess changes in outcomes with both time and date as the x-axis
53. Assess at-risk and not at-risk patients for outcome changes separately
54. Consider hiring a (part-time) data scientist or intern to implement many of these recommendations

RESULTS-TO-PRACTICE

55. Reconsider regimens and emphasis on areas of the intervention based on results
56. Regularly re-assess data analysis methods and approach
