# Supplementary material for: Complex Interventions Deserve Complex Evaluations: A Transdisciplinary Approach to Evaluation of a Preventive Personalized Medicine Intervention
Source: Front Public Health. 2022 Feb 4;10:793137. doi: 10.3389/fpubh.2022.793137 (PMC8854757; doi:10.3389/fpubh.2022.793137)

**DATA SHEET S2**. Graphical results of quantitative analysis

This figure shows the results presented in Table 5. Each outcome is accompanied by two graphs (e.g. A1 and A2): the first shows the model-produced trajectories of the at-risk and not-at-risk populations and the second shows the trajectories of the lower educated, high educated, male, and female populations. These trajectories are not calculated as statistical comparisons to the other consultee groups sharing the plot. Insignificant findings are indicated by a dashed line, and years are indicated by vertical gridlines. Note that many trajectories become unrealistic after some time since Gentest would likely adjust the prescribed intervention after the consultee was no longer considered at-risk.

1. HbA1c

(A1) (A2)


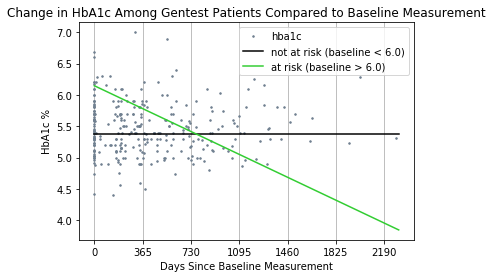

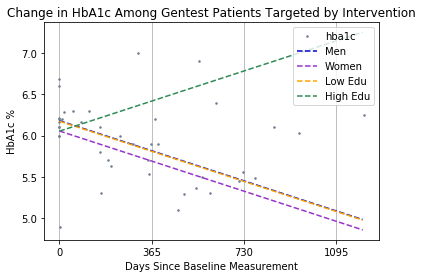


1. Triglyceride

(B1) (B1)


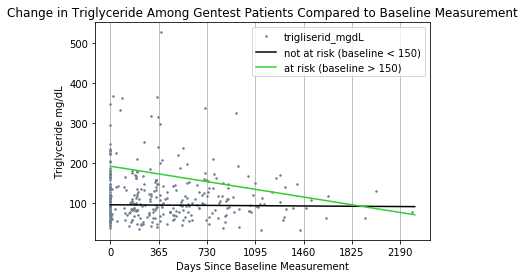

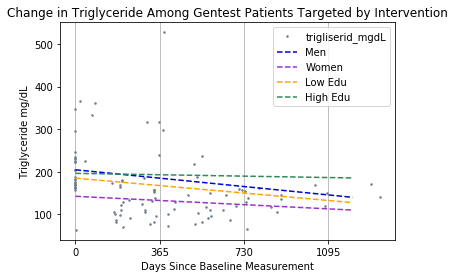


1. Homocysteine

(C1) (C2)

**
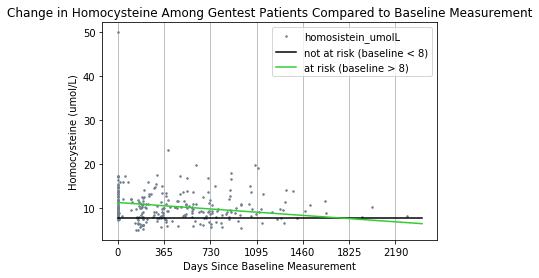

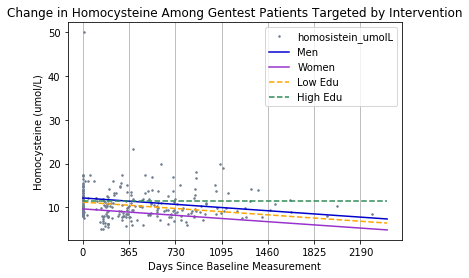
**

1. Magnesium

(D1) (D2)


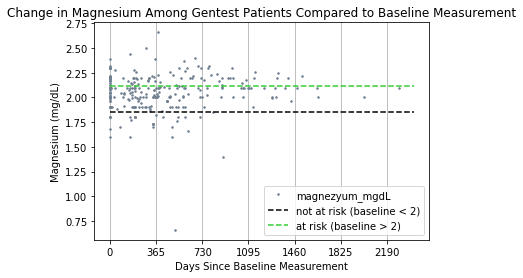

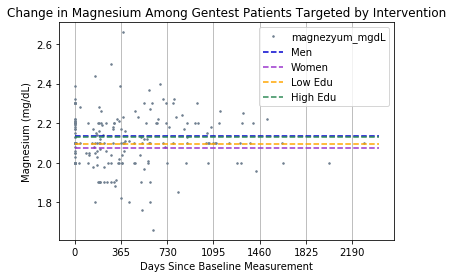


1. Selenium

(E1) (E2)


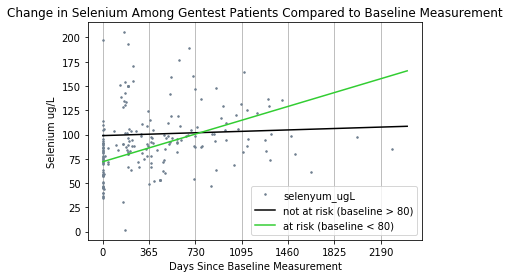

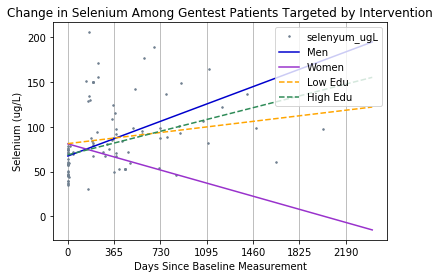


1. Vitamin B12

(F1) (F2)


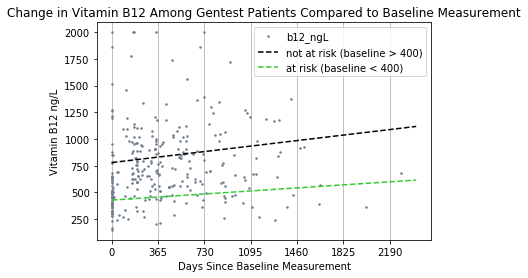

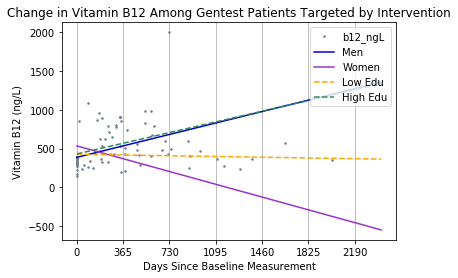


1. Vitamin D

(G1) (G2)


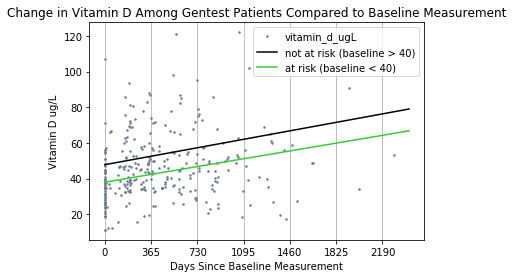

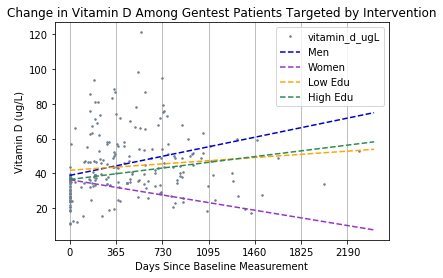


1. High-Sensitive CRP

(H1) (H2)


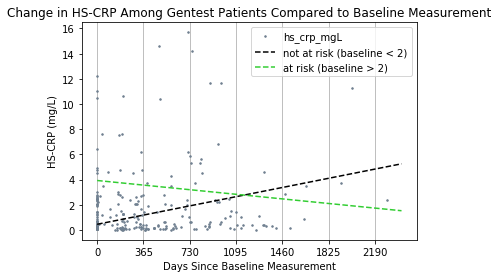

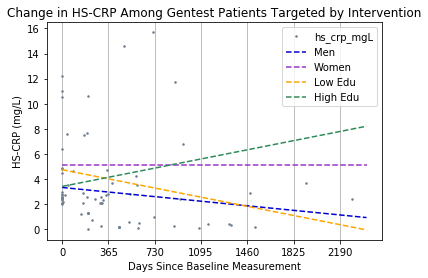


1. Total:HDL Cholesterol Ratio

(I1) (I2)


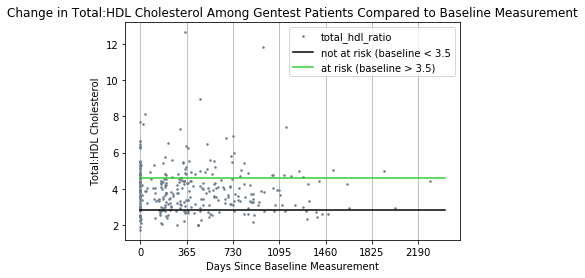

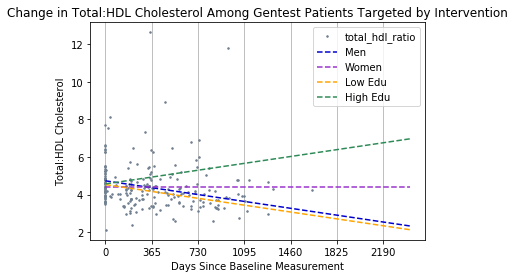


1. BMI

(J1) (J2)


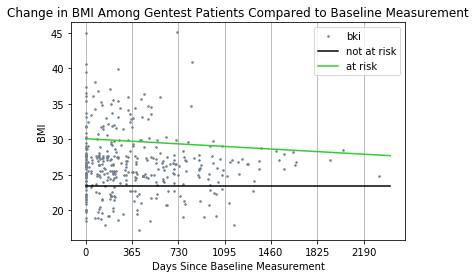

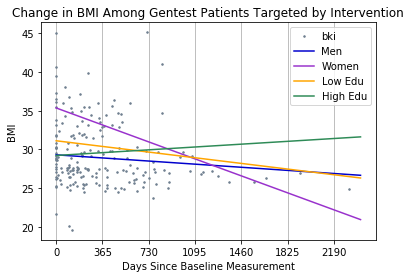


1. Body Fat %

(K1) (K2)


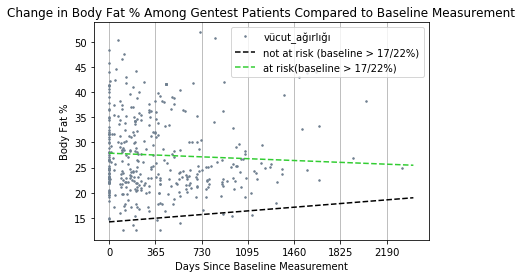

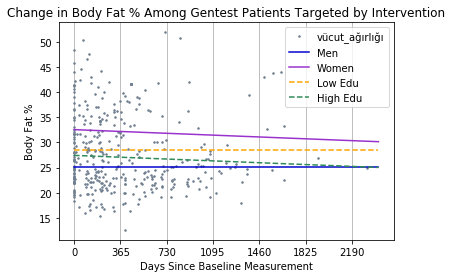


1. Waist:Height Ratio

(L1) (L2)


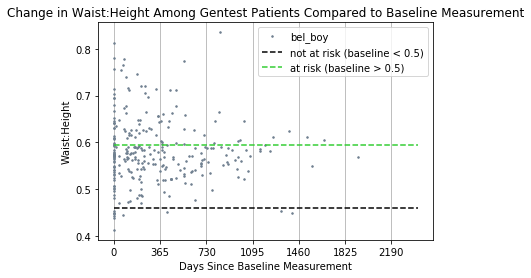

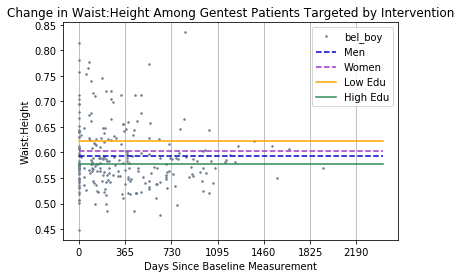


1. Systolic Blood Pressure

(M1) (M2)


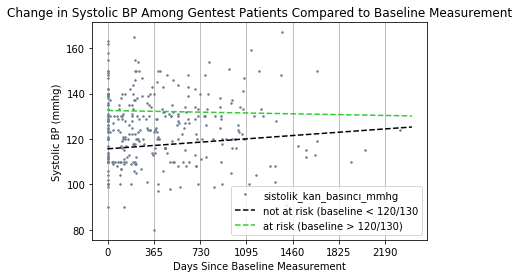

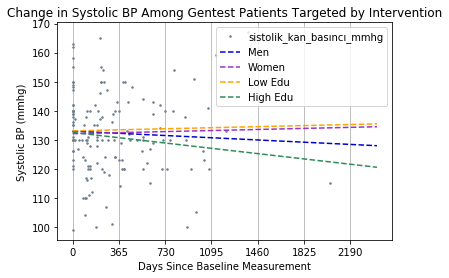


1. Diastolic Blood Pressure

(N1) (N2)


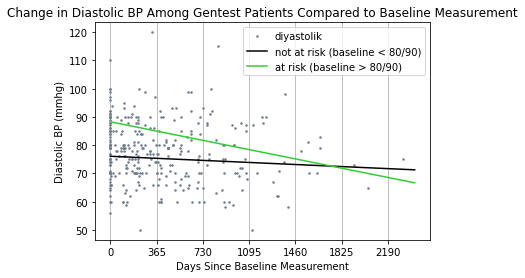

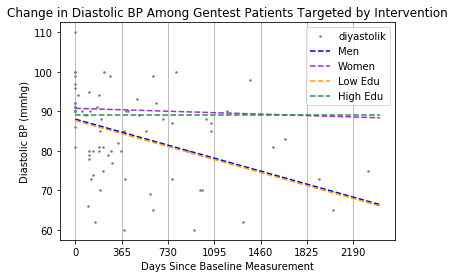

Supplement: Supplementary file 2 [file Data_Sheet_2.docx]
