# Supplementary material for: Complex Interventions Deserve Complex Evaluations: A Transdisciplinary Approach to Evaluation of a Preventive Personalized Medicine Intervention
Source: Front Public Health. 2022 Feb 4;10:793137. doi: 10.3389/fpubh.2022.793137 (PMC8854757; doi:10.3389/fpubh.2022.793137)
Supplement: Supplementary file 3 [file Table_1.docx]

**TABLE S1.** Risk thresholds analysis

| Outcome | Gentest risk threshold | Industry-standard threshold | Alignment |
| --- | --- | --- | --- |
| HbA1c | > 6 | > 5,7 – 6,4 (31) | Similar |
| Triglyceride | > 150 | > 150 (32) | Similar |
| Homocysteine | > 8 | > 15 (33) | Stricter |
| Magnesium | > 2 | > 2,3 (34) | Similar |
| Selenium | < 80 | < 70 (35) | Stricter |
| Vitamin B12 | < 400 | < 200  65+: < 300 (36) | Much stricter |
| Vitamin D | < 40 | < 30 (37) | Stricter |
| HS-CRP | > 2 | > 2 (38) | Similar |
| Total : HDL | > 3,5 | > 5 (39) | Stricter |
| BMI | F: > 28  F50: > 30  M: > 25  M50: > 28 | > 24,9 (40) | Less strict; adjusted for Turkish population specifically |
| Body Fat % | M: > 20%F: > 24% | M: > 23%  W: > 28% (41) | Stricter |
| Waist : Height | > 0,5 | > 0,55 (42) | Similar |
| Blood Pressure | > 120/80  50+: > 130/90 | > 130/80 (43) | Similar |

**REFERENCES:**

31. Li J, Ma H, Na L, Jiang S, Lv L, Li G et al. Increased Hemoglobin A1c Threshold for Prediabetes Remarkably Improving the Agreement Between A1c and Oral Glucose Tolerance Test Criteria in Obese Population. The Journal of Clinical Endocrinology & Metabolism (2015) 100(5):1997-2005.

32. Mayo Clinic. Can triglycerides affect my heart health? (2020). https://www.mayoclinic.org/diseases-conditions/high-blood-cholesterol/in-depth/triglycerides/art-20048186 [Accessed April 29, 2020].

33. University of Rochester Medical Center Homocysteine - Health Encyclopedia (2020). https://www.urmc.rochester.edu/encyclopedia/content.aspx?ContentTypeID=167&ContentID=homocysteine [Accessed April 20, 2020].

34. Mayo Clinic. MGS - Clinical: Magnesium, Serum (2020). https://www.mayocliniclabs.com/test-catalog/Clinical+and+Interpretive/8448 [Accessed April 29, 2020].

35. Mayo Clinic. SES - Clinical: Selenium, Serum (2020). <https://www.mayocliniclabs.com/test-catalog/Clinical+and+Interpretive/9765> [Accessed April 29, 2020].

36. Hanna S, Lachover L, Rajarethinam R. Vitamin B12 Deficiency and Depression in the Elderly: Review and Case Report. The Primary Care Companion to the Journal of Clinical Psychiatry (2009) 11(5):269-270.

37. Harvard Health. Vitamin D and your health: Breaking old rules, raising new hopes (2020). https://www.health.harvard.edu/staying-healthy/vitamin-d-and-your-health-breaking-old-rules-raising-new-hopes [Accessed April 29, 2020].

38. Carrero J, Andersson Franko M, Obergfell A, Gabrielsen A, Jernberg T. hsCRP Level and the Risk of Death or Recurrent Cardiovascular Events in Patients With Myocardial Infarction: a Healthcare‐Based Study. Journal of the American Heart Association (2019) 8:11. doi: [10.1161/JAHA.119.012638](https://dx.doi.org/10.1161%2FJAHA.119.012638)

39. University of Rochester Medical Center. Lipid Panel with Total Cholesterol: HDL Ratio - Health Encyclopedia (2020). https://www.urmc.rochester.edu/encyclopedia/content.aspx?ContentTypeID=167&ContentID=lipid_panel_hdl_ratio [Accessed April 29, 2020].

40. World Health Organization (WHO). Body mass index - BMI (2020). <https://www.euro.who.int/en/health-topics/disease-prevention/nutrition/a-healthy-lifestyle/body-mass-index-bmi> [Accessed March 16, 2020].

41. Ho-Pham L, Campbell L, Nguyen T. More on Body Fat Cutoff Points. Mayo Clinic Proceedings (2011) 86(6):584.

42. Rådholm K, Chalmers J, Ohkuma T, Peters S, Poulter N, Hamet P et al. Use of the waist-to- height ratio to predict cardiovascular risk in patients with diabetes: Results from the ADVANCE-ON study. Diabetes, Obesity and Metabolism (2018) 20(8):1903-1910.

43. Brook R, Rajagopalan S. 2017 ACC/AHA/AAPA/ABC/ACPM/AGS/APhA/ASH/ASPC/NMA/PCNA Guideline for the Prevention, Detection, Evaluation, and Management of High Blood Pressure in Adults. A report of the American College of Cardiology/American Heart Association Task Force on Clinical Practice Guidelines. Journal of the American Society of Hypertension (2018) 12(3):238
