# Supplementary material for: Complex Interventions Deserve Complex Evaluations: A Transdisciplinary Approach to Evaluation of a Preventive Personalized Medicine Intervention
Source: Front Public Health. 2022 Feb 4;10:793137. doi: 10.3389/fpubh.2022.793137 (PMC8854757; doi:10.3389/fpubh.2022.793137)
Supplement: Supplementary file 4 [file Table_2.docx]

**TABLE S2.** Additional Results of Quantitative Analysis

| **Measurement** | **Risk group** | **n** | **%** | **β or Coefficient** | **p** |
| --- | --- | --- | --- | --- | --- |
| **HbA1c** | | | | | |
| **Baseline risk** | Lower educated | 5 | 38% | 6.175 | 0.600 |
|  | High educated | 8 | 62% | 6.054 |  |
|  | Male | 8 | 62% | 6.181 | NA |
|  | Female | 5 | 38% | 6.056 |  |
| **Trend** | Lower educated | 5 | 38% | -0.001 | 0.215 |
|  | High educated | 8 | 62% | -0.001 |  |
|  | Male | 8 | 62% | -0.001 | 0.145 |
|  | Female | 5 | 38% | 0.001 |  |
| **Triglyceride** | | | | | |
| **Baseline risk** | Lower educated | 8 | 32% | 184.791 | 0.720 |
|  | High educated | 17 | 68% | 196.070 |  |
|  | Male | 20 | 80% | 204.27 | 0.074 |
|  | Female | 5 | 20% | 142.1 |  |
| **Trend** | Lower educated | 8 | 32% | -0.048 | 0.832 |
|  | High educated | 17 | 68% | -0.009 |  |
|  | Male | 20 | 80% | -0.054 | 0.695 |
|  | Female | 5 | 20% | -0.027 |  |
| **Homocysteine** | | | | | |
| **Baseline risk** | Lower educated | 30 | 38% | 11.209 | 0.761 |
|  | High educated | 50 | 63% | 11.460 |  |
|  | Male | 52 | 65% | 12.149 | 0.002* |
|  | Female | 28 | 35% | 9.648 |  |
| **Trend** | Lower educated | 30 | 38% | -0.002 | 0.769 |
|  | High educated | 50 | 63% | <0.001 |  |
|  | Male | 52 | 65% | -0.001 | 0.199 |
|  | Female | 28 | 35% | 0.002 |  |
| **Magnesium** | | | | | |
| **Baseline risk** | Lower educated | 24 | 37% | 2.093 | 0.241 |
|  | High educated | 41 | 63% | 2.130 |  |
|  | Male | 45 | 69% | 2.134 | 0.076 |
|  | Female | 20 | 31% | 2.073 |  |
| **Trend** | Lower educated | 24 | 37% | <0.001 | 0.969 |
|  | High educated | 41 | 63% | <0.001 |  |
|  | Male | 45 | 69% | <0.001 | 0.848 |
|  | Female | 20 | 31% | <0.001 |  |
| **Selenium** | | | | | |
| **Baseline risk** | Lower educated | 13 | 34% | 81.173 | 0.234 |
|  | High educated | 25 | 66% | 68.669 |  |
|  | Male | 24 | 63% | 67.347 | 0.052 |
|  | Female | 14 | 37% | 80.900 |  |
| **Trend** | Lower educated | 13 | 34% | 0.017 | 0.203 |
|  | High educated | 25 | 66% | 0.036 |  |
|  | Male | 24 | 63% | 0.053 | 0.030* |
|  | Female | 14 | 37% | -0.040 |  |
| **Vitamin B12** | | | | | |
| **Baseline risk** | Lower educated | 7 | 25% | 426.667 | 0.967 |
|  | High educated | 21 | 75% | 431.080 |  |
|  | Male | 17 | 61% | NA | NA |
|  | Female | 11 | 39% | NA |  |
| **Trend** | Lower educated | 7 | 25% | -0.026 | 0.013* |
|  | High educated | 21 | 75% | 0.377 |  |
|  | Male | 17 | 61% | NA | NA |
|  | Female | 11 | 39% | NA |  |
| **Vitamin D** | | | | | |
| **Baseline risk** | Lower educated | 21 | 34% | 41.857 | 0.271 |
|  | High educated | 40 | 66% | 36.551 |  |
|  | Male | 29 | 57% | 38.902 | 0.552 |
|  | Female | 22 | 43% | 36.432 |  |
| **Trend** | Lower educated | 21 | 34% | 0.005 | 0.162 |
|  | High educated | 40 | 66% | 0.009 |  |
|  | Male | 29 | 57% | 0.015 | 0.062 |
|  | Female | 22 | 43% | -0.012 |  |
| **High Sensitive CRP** | | | | | |
| **Baseline risk** | Lower educated | 12 | 43% | 4.740 | 0.314 |
|  | High educated | 16 | 57% | 3.408 |  |
|  | Male | 19 | 68% | 3.324 | 0.176 |
|  | Female | 9 | 32% | 5.112 |  |
| **Trend** | Lower educated | 12 | 43% | -0.002 | 0.166 |
|  | High educated | 16 | 57% | 0.002 |  |
|  | Male | 19 | 68% | -0.001 | 0.988 |
|  | Female | 9 | 32% | <0.001 |  |
| **Total:HDL Cholesterol Ratio** | | | | | |
| **Baseline risk** | Lower educated | 18 | 39% | 4.532 | 0.906 |
|  | High educated | 28 | 61% | 4.566 |  |
|  | Male | 40 | 70% | 4.731 | 0.067 |
|  | Female | 17 | 30% | 4.416 |  |
| **Trend** | Lower educated | 18 | 39% | -0.001 | 0.172 |
|  | High educated | 28 | 61% | 0.001 |  |
|  | Male | 40 | 70% | -0.001 | 0.499 |
|  | Female | 17 | 30% | <0.001 |  |
| **BMI** | | | | | |
| **Baseline risk** | Lower educated | 20 | 47% | 31.188 | 0.151 |
|  | High educated | 23 | 53% | 29.220 |  |
|  | Male | 36 | 84% | 29.306 | 0.015* |
|  | Female | 7 | 16% | 35.351 |  |
| **Trend** | Lower educated | 20 | 47% | -0.002 | 0.001* |
|  | High educated | 23 | 53% | 0.001 |  |
|  | Male | 36 | 84% | -0.001 | 0.003* |
|  | Female | 7 | 16% | -0.006 |  |
| **Body Fat %** | | | | | |
| **Baseline risk** | Lower educated | 31 | 41% | 28.502 | 0.854 |
|  | High educated | 45 | 59% | 28.808 |  |
|  | Male | 49 | 64% | 25.932 | 0.391 |
|  | Female | 27 | 36% | 36.463 |  |
| **Trend** | Lower educated | 31 | 41% | <0.001 | <0.001* |
|  | High educated | 45 | 59% | -0.001 |  |
|  | Male | 49 | 64% | <0.001 | 0.391 |
|  | Female | 27 | 36% | -0.001 |  |
| **Waist:Height Ratio** | | | | | |
| **Baseline risk** | Lower educated | 28 | 38% | 0.622 | 0.004* |
|  | High educated | 45 | 62% | 0.578 |  |
|  | Male | 52 | 71% | 0.593 | 0.147 |
|  | Female | 21 | 29% | 0.602 |  |
| **Trend** | Lower educated | 28 | 38% | <0.001 | 0.609 |
|  | High educated | 45 | 62% | <0.001 |  |
|  | Male | 52 | 71% | <0.001 | 0.633 |
|  | Female | 21 | 29% | <0.001 |  |
| **Systolic Blood Pressure** | | | | | |
| **Baseline risk** | Lower educated | 17 | 44% | 133.068 | 0.879 |
|  | High educated | 22 | 56% | 132.547 |  |
|  | Male | 26 | 67% | 133.008 | 0.794 |
|  | Female | 13 | 33% | 132.094 |  |
| **Trend** | Lower educated | 17 | 44% | 0.001 | 0.433 |
|  | High educated | 22 | 56% | -0.005 |  |
|  | Male | 26 | 67% | -0.002 | 0.739 |
|  | Female | 13 | 33% | 0.001 |  |
| **Diastolic Blood Pressure** | | | | | |
| **Baseline risk** | Lower educated | 9 | 43% | 87.645 | 0.696 |
|  | High educated | 12 | 57% | 89.132 |  |
|  | Male | 16 | 76% | 88.008 | 0.553 |
|  | Female | 5 | 24% | 90.754 |  |
| **Trend** | Lower educated | 9 | 43% | -0.009 | 0.937 |
|  | High educated | 12 | 57% | <0.001 |  |
|  | Male | 16 | 76% | -0.009 | 0.826 |
|  | Female | 5 | 24% | -0.001 |  |
