# Supplementary figures and images for: Complex Interventions Deserve Complex Evaluations: A Transdisciplinary Approach to Evaluation of a Preventive Personalized Medicine Intervention
Source: Front Public Health. 2022 Feb 4;10:793137. doi: 10.3389/fpubh.2022.793137 (PMC8854757; doi:10.3389/fpubh.2022.793137)

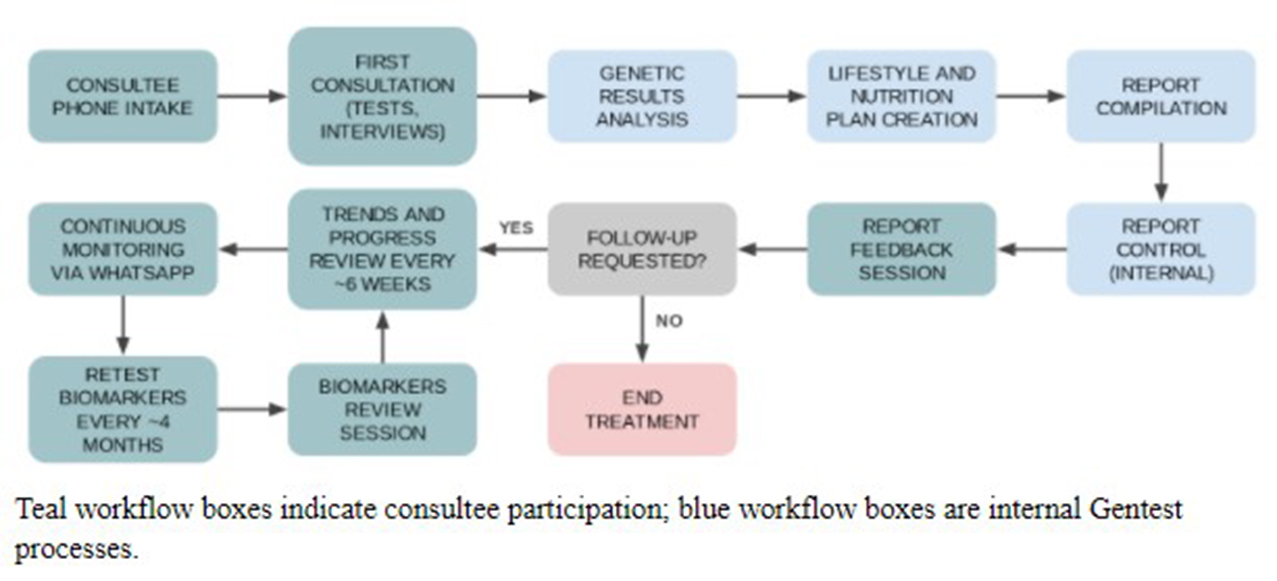

Supplement: Supplementary file 5 [file Figure_1.JPEG]

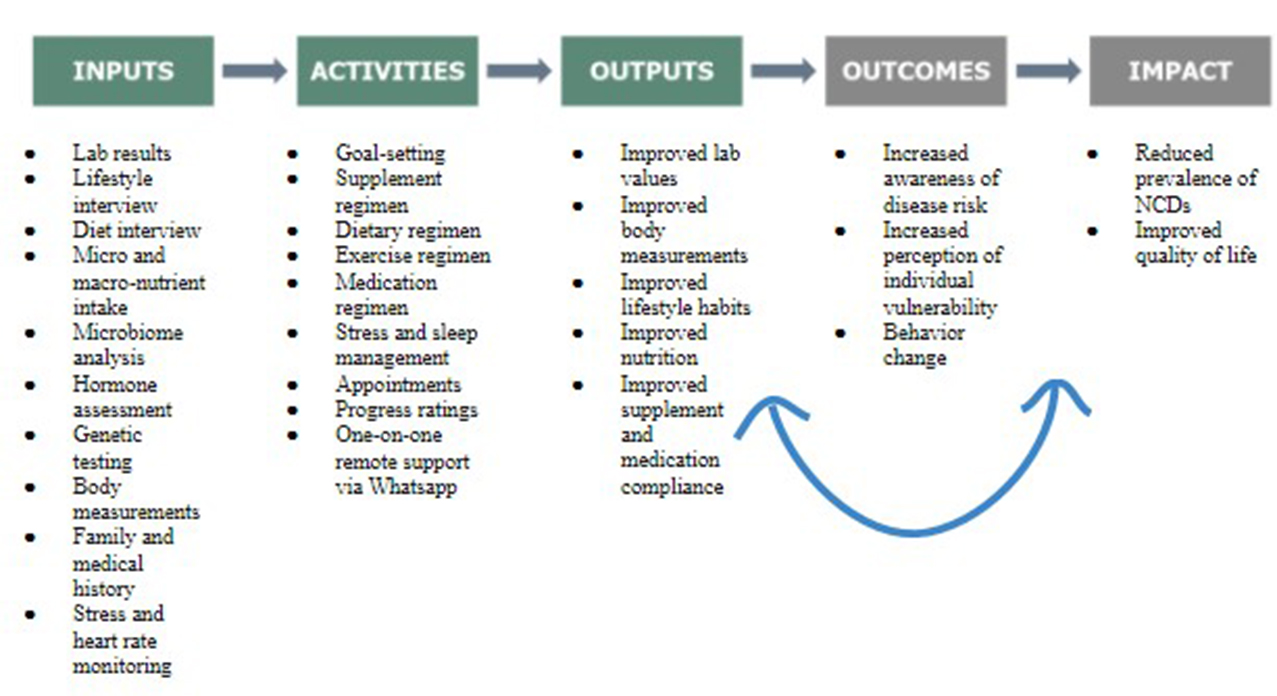

Supplement: Supplementary file 6 [file Figure_2.JPEG]
